# Maritime Response and Emergency Care for Irregular Migrants: Experiences of Spanish Rescue Professionals

**DOI:** 10.3390/healthcare14010123

**Published:** 2026-01-04

**Authors:** María del Mar Jiménez-Lasserrotte, Andrés Pomares Rodríguez, Dulcenombre de María García-López, Verónica Díaz-Sotero, Anabel Chica-Pérez, José Granero-Molina

**Affiliations:** 1 Department of Nursing, Physiotherapy and Medicine, University of Almeria, 04120 Almeria, Spain; mjl095@ual.es (M.d.M.J.-L.); acp819@ual.es (A.C.-P.); jgranero@ual.es (J.G.-M.); 2 Faculty of Health Sciences, University of Almería, 04120 Almeria, Spain; apr701@inlumine.ual.es; 3 Hospital Universitario Poniente, 04700 Almeria, Spain; veronica.diaz.sotero.sspa@juntadeandalucia.es; 4 Facultad de Ciencias de la Salud, Universidad Autónoma de Chile, Temuco 4780000, Chile

**Keywords:** emergency healthcare, irregular migration, maritime rescue, public health emergencies, vulnerability

## Abstract

**Background/Objectives**: Irregular maritime migration is a global health challenge as it combines exposure to extreme physical risks with profound psychosocial vulnerability. Understanding the role of maritime rescue professionals is essential for designing effective public health strategies that respond to these realities. The objective of this study was to explore and describe the experiences of SASEMAR professionals involved in the emergency care and rescue of irregular migrants arriving by small boat to the Spanish coast. **Methods**: Using a qualitative descriptive design, we conducted in-depth interviews and focus groups with 12 SASEMAR professionals. Thematic analysis was performed using Braun and Clarke’s approach with ATLAS.ti software. **Results**: The findings were grouped into three overarching themes: (1) Innovative Emergency Response Systems for Vulnerable Populations; (2) Holistic Health Approaches in Maritime Emergency Care; and (3) Integrated Approaches to Resource Use and Migrant Care Policies. **Conclusions**: Rescuing irregular migrants at sea requires coordinated, flexible, and multidisciplinary strategies to address diverse profiles and challenging conditions. Strengthening rescue capacity and policies is essential to ensure effective, human care during maritime emergencies.

## 1. Introduction

Migration is both an inherent feature of a globalised world and a significant humanitarian challenge [1]. By mid-2024, the number of international migrants was estimated at 304 million, representing 3.7% of the global population [2]. Within this broader context, irregular movements—particularly those involving precarious sea crossings—pose a major public health challenge [3]. The concentration of immediate, life-threatening risks during these journeys exacerbates pre-existing inequalities linked to poverty, structural violence, and limited access to basic resources [4,5]. From the perspective of the social determinants of health [6,7], irregular migration must be understood as a consequence of structural inequities that directly affect the health and well-being of individuals and communities [8].

The European Union currently receives approximately one-third of the world’s migrant population, many of whom cross the Mediterranean Sea to reach the coasts of Greece, Italy, and Spain [2]. Spain has become one of the principal destinations for maritime migration [9], with 63,970 irregular migrants (IMs) arriving by boat in 2024 alone [10]. Although irregular migration to Spain predominantly occurs through regular entry followed by visa overstay, particularly via air travel [11], maritime arrivals by small boat represent a distinct subgroup characterised by extreme vulnerability and acute, life-threatening health risks [3]. The term irregular migrant (IM) refers to “a person who, due to unauthorised entry, failure to comply with an entry condition, or the expiration of their visa, lacks legal status in a transit or host country” [12]. IMs use various routes to escape adverse conditions, and the Central Mediterranean crossing remains among the deadliest [13,14], with more than 29,000 people reported dead or missing at sea since 2014 [13]. Although most people undertaking these journeys are young adult men [15], the number of pregnant women, children, and individuals with disabilities has increased, further complicating rescue operations [16]. These figures capture not only sustained migratory flows but also a global health crisis in which inequality, violence, and extreme vulnerability converge [17,18].

The journey in small boats exposes IMs to multiple health and life-threatening conditions, including hypothermia, dehydration, trauma [16,19], chemical burns [20], obstetric emergencies [21], cardiac arrest, and cardiorespiratory complications [22]. Additional factors such as overcrowding, lack of food and water, and experiences of physical or symbolic violence—both before and during the journey—further compound their vulnerability [23,24,25]. These circumstances create a profound biopsychosocial vulnerability that requires an approach grounded in trauma-informed care [26]. In this scenario, Spain’s Maritime Rescue teams (SASEMAR) constitute the first point of contact for health and humanitarian care [27]. Their role is essential within a system that demands not only immediate clinical responses but also cultural competence, ethical sensitivity, and emotional containment skills [28,29].

Although epidemiological studies on maritime rescues and healthcare provided at ports exist [10,30], little is known about the experiences of the professionals responsible for rescues at sea. Understanding their perspectives sheds light not only on the clinical and logistical challenges they face but also on the ethical and organisational dimensions of rescue operations. From the standpoint of resilient health systems in emergencies [31,32,33], the practices and adaptive strategies implemented by these teams exemplify organisational innovation in contexts of high uncertainty. Moreover, analysing their experiences provides evidence to inform the development of new protocols, public health policies, and intersectoral strategies aimed at ensuring dignified and equitable care for one of today’s most vulnerable populations. Therefore, the objective of this study was to explore and describe the experiences of SASEMAR professionals involved in the emergency care and rescue of irregular migrants arriving by small boat to the Spanish coast.

## 2. Materials and Methods

### 2.1. Design

A descriptive qualitative study was conducted. This design enabled participants’ experiences to be articulated in their own words, without external reinterpretation [34]. This approach ensured fidelity to the data collected while allowing the use of everyday, concrete language [35]. This made it easier to understand the experiences of maritime rescue professionals in health emergencies. The Consolidated Criteria for Qualitative Research Design (COREQ) were followed [36].

### 2.2. Participants and Context

This study took place at the Rescue Coordination Center (RCC) in a province in southern Spain. The participants were recruited through convenience sampling. The inclusion criteria were (1) being a SASEMAR professional; (2) having at least one year of experience; and (3) having experienced first-hand the rescue of IMs arriving in small boats. The exclusion criterion was not wanting to participate in the study. The head of the RCC participated in the recruitment of the sample. The principal investigator personally contacted the participants interested in the study. All participants were informed of the purpose of the study, and informed consent for their voluntary participation was requested. The sociodemographic data of the participants were collected beforehand. Fifteen SASEMAR professionals were invited to participate, but three declined due to scheduling conflicts. The final sample consisted of 12 SASEMAR professionals (Table 1).

### 2.3. Data Collection

Data collection included eight individual in-depth interviews (IDIs) and one focus group (FG) with four participants, conducted at an RCC office between January and March 2025. Both the IDIs and the FG were conducted by several researchers trained in qualitative research, following a script with open-ended questions (Table 2). This script included relevant questions related to the maritime rescue team’s approach to the care and rescue of migrants arriving by boat. At the beginning of each session, sociodemographic data were collected, the study protocol was explained to the participants, and informed consent forms were signed. The interviews and focus groups lasted approximately 57 min. Both the interviews and focus groups were audio-recorded for subsequent transcription and analysis by the research team. In addition, notes were taken on non-verbal aspects of communication. Data collection ended when data saturation was achieved, defined as the point at which no new themes or dimensions emerged from the data. The combination of IDIs and the FG provided complementary insights and facilitated triangulation, enhancing the depth and richness of the analysis.

### 2.4. Data Analysis

Thematic data analysis was carried out using ATLAS.ti.23 software and following the approach proposed by Braun & Clarke [37] for descriptive qualitative studies: (1) complete reading of all transcripts to obtain a general sense of what the participants said; (2) systematic coding of the data, identifying the most representative quotes and assigning codes to reflect their meaning; (3) initial themes were created by grouping codes with shared meanings and linking them to a central idea (Table 3); (4) the themes were developed and reviewed to check their consistency and define the results of the research; (5) the themes were refined, defined and named; and (6) the final report was written.

### 2.5. Rigour

The rigour criteria defined by Lincoln & Guba [38,39] were followed. The researchers had extensive experience in qualitative and migration research, ensuring the credibility of the study. For reliability, a data triangulation strategy was used to verify the information collected by several researchers. In addition, a detailed description of the objective and methodology was provided. The experience and context of the study were described in detail, taking into account the experiences of the participants, as well as the location of the study. Transferability was ensured due to the quality of the methodological approach. The study may be replicated in future research. In addition, the participants’ narratives were described until data saturation was achieved. For confirmability, the data analysis included verbatim excerpts from the study participants, who confirmed the interpretation of these direct quotes.

### 2.6. Ethical Considerations

This research was conducted in accordance with the ethical principles of the Declaration of Helsinki [40] and Organic Law 3/2018 of 5 December on the Protection of Personal Data and Guarantee of Digital Rights. Permission was obtained from the Ethics Committee of the University of Almería with the following protocol number: EFM 299/24. The participants were informed that their participation was voluntary, anonymous, and confidential, and that the information obtained would be used exclusively for research purposes. To this end, those interested in participating signed an informed consent form, bearing in mind that they could withdraw from the study at any time if they so choose, or even refrain from answering any of the questions if they wished.

## 3. Results

The final sample consisted of 12 SASEMAR professionals with an average age of 45.7 years and an average length of service of 13.7 years. Three themes and six subthemes were developed from the data analysis (Table 4). These themes and subthemes provided insight into the experiences of SASEMAR professionals in providing emergency care and rescue to IMs arriving by small boat.

### 3.1. Innovative Emergency Response Systems for Vulnerable Populations

This theme addresses the Spanish Maritime Rescue System’s ability to provide an immediate and flexible response to emergencies through multi-level coordination and the use of location technologies. However, the participants highlighted how operational effectiveness also depends on interdisciplinary communication and the cultural competence of staff. This is essential for providing safe care tailored to the needs of migrants.

#### 3.1.1. Adaptive Multi-Level Coordination Protocols

The Spanish Maritime Rescue System (SASEMAR) operates under a model of permanent coordination, which is active 24 h a day, seven days a week. This scheme guarantees the constant presence of controllers and on-call teams, ensuring an immediate response to any maritime emergency. The participants covered the geographical area between Almuñécar and Águilas, including the maritime borders with Morocco and Algeria. Although each centre is responsible for its own area, the professionals indicated that they could request support from other areas in emergency situations or when close by.

*“I manage what happens in my area, and what happens in the other area is the competence of someone else.”* 

(FG1-1)

In this context, operational flexibility becomes essential, as it frequently requires moving beyond formal boundaries and administrative limits to prioritise saving lives. The participants highlighted that immediacy was the primary concern, and for that reason, when a nearby vessel was able to respond more quickly, resources were reallocated accordingly, ensuring that intervention was not delayed by bureaucratic procedures.

*“When one of our vessels can get there first, we assume responsibility for the emergency, even if it is not in our area.”* 

(FG1-2)

*“We talk to the neighbouring centre to see if they can get there first, because sometimes it’s a matter of minutes and that can make all the difference to the outcome of the rescue.”* 

(FG1-3)

Rescue priorities are structured around established vulnerability criteria, whereby pregnant women, children, and migrants with disabilities are given precedence. Interventions are subsequently adapted in accordance with the clinical severity of each case, the condition of the vessel involved, and prevailing weather conditions. Within this process, the degree of preparation undertaken before the journey emerges as a critical factor. The professionals explained that while some groups of IMs arrived with life jackets and basic navigation equipment, others reached rescue teams in extremely precarious circumstances, therefore requiring immediate care.

*“The sub-Saharan Africans tend to arrive in much more precarious conditions. They are the group most in need of care from rescue personnel.”* 

(FG1-4)

Technological advances have radically transformed rescue detection and coordination. The use of satellite phones, GPS, and surveillance systems allows vessels to be located more quickly and accurately. On some occasions, the IMs themselves contacted the rescue services directly and provided their exact location, speeding up response times. This early warning network is complemented by information provided by merchant ships, family members and coastal authorities. In addition, simulation tools, such as ‘IBISAR’, are crucial in anticipating the drift of small boats and planning the allocation of resources in advance.

*“Now it’s much easier. If they come equipped with satellite phones, they send you their location, or they even call you themselves.”* 

(IDI-3)

Despite these advances, professionals pointed out that there is still a great deal of uncertainty, and that every operational decision involves a high level of responsibility. This is how one of the participants described it:

*“Even if you have the exact location, you never know what you’re going to find. When you arrive, the lives of those people depend on how fast you’ve been.”* 

(IDI-7)

Coordination, however, does not only stem from formal orders from headquarters, but depends on the ability to adjust decisions in real time between different operational levels, as participants pointed out:

*“You may have orders from above, but if the captain says the conditions at sea are impossible, you have to find another solution. It’s about coordination at all levels, not just what the centre says.”* 

(IDI-8)

#### 3.1.2. Interdisciplinary Communication and Cultural Competency

The first step in initiating the rescue of IMs is to identify what type of care they require as quickly as possible. When an alert call is received, controllers act quickly and efficiently, coordinating with other professionals. According to the participants, they respond to the situation by assuming the worst possible scenario while analysing and verifying whether there is a real danger.

*“When an alert comes in, you have to analyse it, gather data and screen it to find out whether it is real or not. Often, the migrant has already reached land, and it is a waste of resources when others may need them.”* 

(IDI-2)

According to the participants, the effectiveness of the rescue also depended on distributed coordination, in which each professional adjusted their decisions based on the information available and the behaviour of the IMs. This implies a distribution of responsibilities among the different hierarchical levels and professionals involved.

*“You have to understand how they react, what they need, and coordinate at all levels.”* 

(IDI-6)

Language barriers posed a constant challenge during rescues, as migrants spoke English, French, Arabic, or other languages. To overcome these barriers, teams implemented multilingual strategies, relying on crew members with language skills or pre-recorded messages. These tools are essential for conveying critical instructions and reassuring IMs in distress. This guarantees an immediate sense of safety and facilitates a better understanding of behaviours and needs.

*“The state of nerves, adrenaline and eagerness to get on board makes it difficult to concentrate, but these are normal feelings. Once they are on board, they calm down and we can talk to them.”* 

(FG1-3)

Interdisciplinary coordination spans from initial detection to safe disembarkation, and involves the National Police, Civil Guard, NGOs and healthcare professionals. This cooperation demands ongoing communication and flexibility to adapt to unforeseen events and changing situations. It is crucial to interpret the cultural and emotional cues of the IMs to ensure their safety and well-being throughout the rescue process.

*“We usually wait until someone from the police or Civil Guard is at the dock to assess the migrants. Until the National Police arrive, no one gets off the boat. In some cases, we have arrived at port and, when the police were not there, they ran away.”* 

(IDI-8)

### 3.2. Holistic Health Approaches in Maritime Emergency Care

Irregular migration by sea exposes IMs to a lethal combination of pre-existing vulnerabilities and extreme conditions, increasing the risk of physical and psychological harm. This theme focuses on how rescue teams should provide comprehensive care that addresses both medical emergencies, such as hypothermia or dehydration, and the emotional impact of trauma.

#### 3.2.1. Social Determinants of Health Among Irregular Migrants

The IMs who arrive at the coast are in a vulnerable condition as a result of their life trajectories, defined by years of poverty, exposure to violence, and hostile environments. These pre-existing factors are exacerbated during the journey in small boats, where factors such as extreme cold, humidity, overcrowding, and lack of food increase the risk of acute health problems. The participants reported cases of hypothermia, dehydration, and trauma. The rescue teams identified high-risk groups and tailored care to their individual needs, prioritising protection and immediate comprehensive care.

*“They all get here thirsty and cold. When pregnant women, children, or people with disabilities arrive, they’re our priority so we can give them holistic care.”* 

(IDI-6)

*“Dehydration from drinking salt water is common. Many of them tell you that they feel nauseous and also have a stomach ache.”* 

(IDI-2)

Moreover, some IMs feign injuries or symptoms to obtain priority care when rescued, which adds complexity to the initial screening. The professionals explained how they must ascertain the real condition of each person at a biopsychosocial level to ensure equal care for all.

*“They are usually in a great hurry. They also sometimes lie out of desperation, claiming to have an emergency that is later not confirmed. For example, someone who had claimed not to be able to move their legs during the rescue began to move them as soon as they reached the boat.”* 

(IDI-1)

The participants noted that the prior experience and resilience of the IMs influenced the care provided during the rescue. Migrants with prior knowledge of navigation, communication, or survival tended to collaborate more actively, which optimised intervention times and reduced risks for everyone on board.

*“Some know how to swim, some have travelled before and understand how they should behave on the boat.”* 

(IDI-3)

The extreme conditions of the journey, combined with social and environmental factors, directly influences the planning and execution of maritime rescues. To ensure effective and timely care, a comprehensive approach is required that assesses vulnerability, physical risk, and the conditions at sea, together with an analysis of the migrants’ behaviour.

*“In every rescue, we must decide who needs immediate care and how to organise resources. All this without forgetting that the rest of them also need care and without underestimating other aspects of their wellbeing.”* 

(IDI-5)

#### 3.2.2. Trauma-Informed Emergency Care Protocols

Rescue efforts include physical and psychological care. Medical care focuses on immediate conditions, such as hypothermia, burns, dehydration, and crush injuries, as well as addressing the emotional trauma of the situation. Medical protocols range from first aid to paediatric care and delivering babies at sea. In addition, multilingual communication strategies are used to convey calm and build trust.

*“In situations involving childbirth on board, we apply specific procedures for physical care and psychological support. For example, in a recent rescue, a mother gave birth and I made sure to hand the baby to her straight away.”* 

(FG1-2)

The study participants highlighted extreme cold as the main cause of death, as it quickly triggers hypothermia and, in some cases, cardiorespiratory arrest. Another lethal threat identified was entrapment, which can occur when the boat suffers damage and migrants are trapped under the weight of those struggling to survive. These are extremely traumatic situations for which the rescue team must be properly trained.

*“Many of them (IMs) cannot swim. When faced with critical situations on the boat, they become desperate and their survival instinct takes over. These are very complex scenarios and we must act without hesitation.”* 

(FG1-2)

Rescue teams face extreme situations, such as half-sunken boats, people stranded for many hours, or dead bodies on board. These harrowing circumstances require specific procedures to ensure dignified care and psychological support. According to the participants, care must be both physical and emotional, as addressing both aspects is essential to mitigate the impact of trauma and prevent further complications.

*“These are very intense rescues. Some people have been at sea for hours and, on occasion, there are fatalities. Part of our job is to manage the emergency while continuing to provide psychological support to the most vulnerable.”* 

(FG1-3)

These human losses can be extremely distressing, such as in the case of babies who have died as a result of hypothermia or certain extreme cultural beliefs. In these circumstances, rescue personnel must provide specific psychological support to mothers and family members. They must guarantee emotional support and respectful care in these highly traumatic circumstances.

*“The most difficult thing is not only the medical emergency itself, but how to comfort families when they lose a baby. There are cultures where they believe that crying brings bad luck, and our job is to accompany them and offer support.”* 

(IDI-6)

### 3.3. Integrated Approaches to Resource Use and Migrant Care Policies

The provision of healthcare in maritime rescues is marked by a constant tension between the need for standardised protocols and the reality of operational improvisation. Although SASEMAR personnel have access to medical radio consultation, they face critical limitations, such as a lack of uniform healthcare training and a shortage of human resources. This issue highlights the urgent need to integrate care, logistics, and institutional frameworks in order to overcome dependence on improvised solutions and ensure an effective humanitarian response.

#### 3.3.1. Resource Optimisation in Emergency Healthcare Delivery

SASEMAR professionals require medical training to manage emergencies that arise during maritime rescue operations. However, such training is not standardised across all crew members; only the captains receive instruction in the use of semi-automatic defibrillators. This represents a substantial limitation in critical scenarios, as skippers are simultaneously responsible for navigating the vessel, thereby constraining their ability to provide timely medical intervention.

*“We have a defibrillator on board, but only the skipper receives training. If I am steering the boat, I cannot attend to the migrants. I do not understand why the others do not receive the same training.”* 

(IDI-8)

During the transfer, rescue crew members can consult any medical emergency via medical radio, a 24 h service that allows them to receive direct instructions from a doctor. The participants explained that all vessels were equipped with standardised first aid kits designed so that, with basic medical knowledge, the crew could apply medical instructions. The role of the remote doctor is crucial, as they also assess whether the situation requires urgent evacuation by helicopter or whether it can be resolved on board while the sea transfer is taking place.

*“If you need to administer any type of medication or have any health-related questions, a radio-medical consultation is carried out. The doctor, who is based in Madrid, assesses all medication. All ships are required to carry the same medication.”* 

(FG1-3)

*“Almost all the women who have given birth are taken away by helicopter, as are the most serious emergencies. We do not have sufficient knowledge to deal with something like that.”* 

(IDI-6)

The participants highlighted the operational limitations resulting from insufficient human resources on a number of occasions. To address these constraints, creative strategies were implemented, such as the collective organisation of infant care in blankets, which freed up staff and ensured the effective continuity of the rescue effort.

*“You put the babies that come, four or five of them, on a blanket and choose one person to look after them. That way, you don’t need four or five people holding one in each arm.”* 

(IDI-5)

A further challenge is posed by situations involving IMs with disabilities, exacerbated by the shortage of crew members. In these cases, IMs in better physical condition are asked to assist in rescuing others with reduced mobility or blindness. Although this practice is operational at the time, it highlights the lack of formal protocols for assisting people with disabilities and highlights a structural dependence on the spontaneous cooperation of IMs.

*“We have received people in wheelchairs, blind people and those with impaired mobility. When the rest of the migrants are physically fit, you always tell them to stay behind so they can help the others.”* 

(FG1-3)

#### 3.3.2. Policy Framework for Integrated Migrant Care

The effective allocation of crew, vessels, helicopters, and technological systems is key to reducing response times and thus increasing the likelihood of rescue. In practice, optimising resources involves not only deploying the available resources but also improvising solutions that enable operational sustainability to be maintained in high-demand contexts.

*“The boats in the most precarious situations are rescued first; you always go for the vulnerable ones first.”* 

(IDI-1)

This decision-making is inherently dynamic and requires balancing clinical urgency with logistical feasibility. The participants described how it is necessary to react quickly, but avoid over-utilising highly costly and limited resources:

*“You have to assess whether the emergency is sustainable enough to get you safely to port. We sometimes lack sufficient resources and cannot afford to make any mistakes.”* 

(FG1-4)

*“They demand that we rescue them quickly. They always say that one of them is in a very bad way, even that they are not breathing. We have to be absolutely sure that this is the case.”* 

(IDI-2)

According to rescue professionals, the lack of clarity in the division of inter-institutional responsibilities creates uncertainty in the transfer of functions, forcing them to resort to improvised decisions. The participants pointed out that this lack of precision highlights the urgent need to establish policies that integrate operational procedures with health, social and legal support following the rescue.

*“In many rescue operations, we realise that the medical staff do not know what information they need to record, and we end up doing the follow-up ourselves.”* 

(IDI-7)

The absence of shared protocols led to inequalities in initial care and increased confusion at critical moments. Therefore, the creation of integrated information systems was a key requirement for the participants, as it ensured continuity of care and optimised inter-institutional coordination.

*“When there is coordination, it reduces tension at the port and we can focus on providing direct care to those who have been rescued.”* 

(FG1-3)

In the words of the professionals themselves, this lack of clarity forced each professional to apply their own criteria, which resulted in significant operational and legal uncertainty:

*“In some cases, no one tells us exactly what to do, and everyone applies their own judgement. This causes confusion, and we do not always know if we are doing the right thing.”* 

(IDI-4)

## 4. Discussion

The aim of this study was to understand and describe the experiences of SASEMAR professionals in providing emergency care and rescue to IMs arriving by boat. The qualitative design allowed us to understand the experiences of these professionals and how they adapt their actions according to the health needs of the IMs and urgency of the rescue operation. The increase in irregular migration from North Africa to the Spanish coast, mainly via the Central Mediterranean route [1], is a phenomenon with clear seasonal patterns in migratory flows [41]. In this context, SASEMAR, in coordination with the Civil Guard, assumes responsibility for ensuring maritime safety in the corresponding jurisdictional waters. While previous studies identify logistical challenges and health vulnerabilities specific to these populations [3,42], the results of this study highlight three critical dimensions: operational adaptability in the face of structural fragility, comprehensive trauma management, and the urgent need for an inter-institutional regulatory framework. These results incorporate the perspective of rescuers as key frontline responders, as previous studies have pointed out [43].

The identification of people in danger often depends on alerts and the limited interpretation of radar images, which leads to a partial understanding of real situations [44]. Unlike other rigid hierarchical or geographical models, SASEMAR operates under permanent and flexible coordination, prioritising the protection of human life [45]. However, the participants’ accounts suggest that such flexibility frequently compensates for structural shortcomings rather than reflecting an adequately resourced system. The shortage of human resources means that improvisation is necessary, often requiring the collaboration of the migrants themselves [46]. Rather than isolated operational issues, these practices reveal structural fragility and the lack of formalised protocols for highly complex rescue scenarios [47]. Added to these challenges is the language barrier, which hinders communication with IMs and limits professionals’ ability to fully assess needs during rescue [3]. This constraint reinforces reliance on rapid, experience-based judgement, particularly under conditions of high demand for immediate healthcare, even in cases not posing an imminent risk to life [16].

In line with international humanitarian protocols, SASEMAR prioritises the care of sick individuals, children, and pregnant women [10]. Nevertheless, the findings reveal significant ethical tensions inherent in triage and prioritisation during maritime rescue. Situations in which some individuals feign or exaggerate injuries to obtain immediate care generate feelings of anxiety and uncertainty among professionals and may increase operational risks for the rescue team [48,49]. These dynamics place rescuers in morally demanding positions, where clinical decision-making intersects with ethical responsibility under conditions of scarcity [48,50]. Importantly, such situations should not be interpreted as individual misconduct, but rather as expressions of extreme vulnerability shaped by prolonged hardship and fear during the migratory journey [51,52].

Consistent with the previous literature, many IMs arrive in highly vulnerable conditions, including physical exhaustion, malnutrition, and trauma [53]. Responding to their needs, therefore, requires not only an immediate physical intervention but also effective management of fear, distress, and disorientation [54,55]. Although comprehensive, trauma-informed approaches are widely advocated in humanitarian settings [56], the maritime environment—marked by instability, restricted mobility, and continuous environmental exposure—intensifies vulnerability and limits the scope and duration of feasible interventions [57].

Beyond clinical and logistical challenges, there is a cumulative emotional and moral burden experienced by SASEMAR professionals. Continuous exposure to human suffering, high-stakes decision-making, and perceived institutional insufficiency contributes to stress, uncertainty, and moral distress [58,59]. While similar experiences have been reported among frontline responders in other humanitarian crises [27], this dimension remains underexplored in maritime rescue research, highlighting a gap between institutional expectations and operational realities.

### 4.1. Strengths and Limitations

This study has several limitations that should be taken into account when interpreting the findings. Although the sample included a diverse group of participants in terms of professional roles and years of experience, all of them were based at a single SASEMAR station located in a southern province of Spain. This limits the extent to which the results can be considered representative of other national or international bases, which may operate under different procedures or encounter distinct challenges. Moreover, the qualitative design of the study enables an in-depth understanding of professionals’ experiences yet inherently restricts the generalisability of the results. In addition, participation among RCC professionals was limited to those who were available and willing to take part, which may have introduced selection bias and limited the representativeness of the findings. However, all professionals working on the rescue vessel operating in the province were included.

Despite these limitations, the study presents several notable strengths. The richness of the data generated through in-depth interviews allowed for the identification of subtle aspects of daily practice and decision-making in high-pressure maritime rescue contexts. The heterogeneity of the participant group facilitated the examination of multiple perspectives within the rescue team. Conducting the interviews in an environment familiar to the professionals encouraged openness and sincerity, contributing to the depth and authenticity of the accounts. Additionally, time dedicated to building rapport before the interviews helped foster trust, enabling participants to express complex experiences related to rescue operations and healthcare provision at sea.

### 4.2. Policy and Practice Implications

The findings of this study underscore the need to move beyond individual-level resilience and address structural responsibility in maritime rescue operations. Caring for particularly vulnerable groups requires additional resources as well as stronger inter-institutional coordination [60,61]. Although SASEMAR personnel receive first-aid training and have access to satellite communication systems that connect them with military hospitals to prioritise interventions and facilitate urgent transfers [62,63], the absence of standardised protocols and integrated information systems remains a critical limitation. Strengthening staff capacity, promoting multidisciplinary and culturally competent training, establishing agreed-upon operational protocols, and developing mechanisms for post-rescue follow-up emerge as key priorities to support both professional well-being and the dignity of IMs [64,65].

In this regard, the emerging literature on information-seeking and digital resilience among refugee populations demonstrate that such resources play a crucial role in longer-term well-being and continuity of care [66]. These perspectives complement the present findings that extend beyond the moment of rescue and support IMs’ navigation of health and social systems after disembarkation. As highlighted by Theodosopoulou et al. [27], the optimisation of available resources and the effective management of priorities during mass-rescue situations are not merely technical challenges, but essential conditions for ethically sound and sustainable emergency response.

## 5. Conclusions

The care of irregular migrants at sea is a complex task that requires comprehensive coordination, operational flexibility, and adaptive strategies capable of responding to diverse migration profiles as well as climatic, health, and social challenges. On the Spanish coast, this rescue process is carried out in an organised and structured manner, with the involvement of specialised professionals whose primary objective is to safeguard the lives of all persons at risk or in distress at sea. Nevertheless, the irregular nature of transit and the precarious conditions in which migrants travel significantly complicate rescue operations. The main challenges encountered by SASEMAR personnel include locating vessels, assessing the health status of those rescued, managing adverse weather conditions, and providing appropriate care to particularly vulnerable groups. These findings highlight the need to guarantee high-quality care through a multidisciplinary approach. The central role of SASEMAR professionals in optimising healthcare provision reinforces the importance of policies and programmes aimed at strengthening their response capacity, ensuring humane and effective care in maritime emergency contexts.

## Figures and Tables

**Table 1:** Sociodemographic characteristics of the participants (N = 12).

Participants	Gender	Age	Nationality	Work Experience (Years)	Job Title
**FG1**					
FG1-1	M	52	Spanish	22	Mechanic
FG1-2	M	54	Spanish	12	Sailor
FG1-3	M	34	Spanish	2	Sailor
FG1-4	M	50	Spanish	18	Captain
**IDI**					
IDI-1	F	35	Spanish	2	Coordinator
IDI-2	F	48	Spanish	8	Coordinator
IDI-3	F	50	Spanish	14	Coordinator
IDI-4	M	47	Spanish	9	Coordinator
IDI-5	M	51	Spanish	23	Mechanic
IDI-6	M	46	Spanish	28	Sailor
IDI-7	M	33	Spanish	7	Sailor
IDI-8	M	49	Spanish	20	Captain

Bold text within the “Participants” column indicates the data collection technique. FG = focus group; IDI = in-depth interview; F = female; M = male.

**Table 2:** Interview protocol.

Phase	Theme	Content/Example Question
Presentation	Purpose	To understand and describe the experiences of SASEMAR professionals in providing emergency care and rescue to IMs arriving by boat.
	Ethical considerations	Voluntary participation, informed consent, possibility of withdrawal, and confidentiality.
Opening	Opening question	Could you tell me about your experiences with IM rescues at sea?
Development	Specific questions	What information do you need to mobilise a rescue unit?
		Who is involved in the rescue process?
		What health-related information do you collect when a rescue is requested?
		How do you decide which actions to prioritise during a rescue?
Closing	Final question	What difficulties do you encounter, and how could the care be improved? Is there anything else about your experience in migrant recues that you would like to share?
	Acknowledgements	Thank you for your time. We are at your disposal should you have any further queries.

**Table 3:** Example of coding strategy.

Quotation	Initial Code	Subtheme	Main Theme
“You have to understand how they react, what they need, and coordinate on all levels” (IDI-6).	Alert, emergency, need, cultural approach	3.1.2. Interdisciplinary Communication and Cultural Competency	3.1. Innovative Emergency Response Systems for Vulnerable Populations

**Table 4:** Theme, subtheme, and condensed meaning units.

Theme	Subtheme	Units of Meaning
Innovative Emergency Response Systems for Vulnerable Populations	Adaptive Multi-Level Coordination Protocols	Flexibility, communication, improvisation, hierarchy, responsiveness, prioritisation.
	Interdisciplinary Communication and Cultural Competency	Sensitivity, language, barriers, empathy, cultural mediation, trust building.
Holistic Health Approaches in Maritime Emergency Care	Social Determinants of Health among Irregular Migrants	Patient-centred communication, food insecurity, health inequity, environmental exposure.
	Trauma-Informed Emergency Care Protocols	Stabilisation strategies, stress reactions, resilience support, emotional safety, fear management.
Integrated Approaches to Resource Use and Migrant Care Policies	Resource Optimisation in Emergency Healthcare Delivery	Resource allocation, logistical efficiency, equipment limitations, time constraints, triage management, supply chain, efficiency.
	Policy Framework for Integrated Migrant Care	Institutional fragmentation, care continuity, health equity policies, protocol standardisation, migrant inclusion.

## Data Availability

The data presented in this study are available on request from the corresponding author due to privacy.
